# The GPR139 reference agonists 1a and 7c, and tryptophan and phenylalanine share a common binding site

**DOI:** 10.1038/s41598-017-01049-z

**Published:** 2017-04-25

**Authors:** Anne Cathrine Nøhr, Willem Jespers, Mohamed A. Shehata, Leonard Floryan, Vignir Isberg, Kirsten Bayer Andersen, Johan Åqvist, Hugo Gutiérrez-de-Terán, Hans Bräuner-Osborne, David E. Gloriam

**Affiliations:** 1 Department of Drug Design and Pharmacology, University of Copenhagen, Universitetsparken 2, 2100 Copenhagen, Denmark; 2 Department of Cell and Molecular Biology, Uppsala University, Biomedical Center, Box 596, SE-751 24 Uppsala, Sweden; 3 Department of Chemistry and Applied Biosciences, ETH Zürich, Vladimir-Prelog-Weg 1-5/10, 8093 Zurich, Switzerland

## Abstract

GPR139 is an orphan G protein-coupled receptor expressed in the brain, in particular in the habenula, hypothalamus and striatum. It has therefore been suggested that GPR139 is a possible target for metabolic disorders and Parkinson’s disease. Several surrogate agonist series have been published for GPR139. Two series published by Shi *et al*. and Dvorak *et al*. included agonists 1a and 7c respectively, with potencies in the ten-nanomolar range. Furthermore, Isberg *et al*. and Liu *et al*. have previously shown that tryptophan (Trp) and phenylalanine (Phe) can activate GPR139 in the hundred-micromolar range. In this study, we produced a mutagenesis-guided model of the GPR139 binding site to form a foundation for future structure-based ligand optimization. Receptor mutants studied in a Ca^2+^ assay demonstrated that residues F109^3×33^, H187^5×43^, W241^6×48^ and N271^7×38^, but not E108^3×32^, are highly important for the activation of GPR139 as predicted by the receptor model. The initial ligand-receptor complex was optimized through free energy perturbation simulations, generating a refined GPR139 model in agreement with experimental data. In summary, the GPR139 reference surrogate agonists 1a and 7c, and the endogenous amino acids l-Trp and l-Phe share a common binding site, as demonstrated by mutagenesis, ligand docking and free energy calculations.

## Introduction

G protein-coupled receptors (GPCRs) constitute the largest family of cell surface proteins. The human genome contains approximately 800 GPCR genes^1^. GPCRs are involved in a broad spectrum of (patho)physiological processes^2, 3^ related to e.g. vision, neurotransmission, immune responses, and metabolism. GPCRs also constitute one of the most important drug target families as about a third of all approved drugs on the market today target a GPCR^4, 5^. Intriguingly, 121 GPCRs are non-sensory orphan receptors^6^ (having unknown endogenous ligands) that could represent yet untapped targets for novel treatments^7^.

GPR139 is a class A orphan GPCR^8^ and its mRNA is predominantly expressed in the striatum, habenula and hypothalamus^9–12^. Attempts to determine the GPR139 protein expression with radioligands have failed^9, 13^. Consistent cross-species expression of GPR139 mRNA in the striatum^9–11, 14^ suggests that GPR139 may play a role in locomotor activity. This is also supported by Liu *et al*. who showed that activation of GPR139 with a surrogate agonist **7c** (also known as JNJ-63533054) leads to decreased spontaneous locomotion activity in rats^9^. One major locomotion pathophysiology is Parkinson’s diseases. MPP^+^ is a toxin used in animals, which produce the neurological defects observed in Parkinson’s patients by degenerating dopaminergic neurons. A recent study by Andersen *et al*. showed that GPR139 agonists protect primary dopaminergic neurons against MPP^+^ *in vitro* ^15^. Based on these findings, GPR139 has been hypothesized as a potential target for the treatment of diseases with impaired movement control, e.g. Parkinson’s disease.

Furthermore, the GPR139 mRNA expression in hypothalamus and habenula suggests a role in the regulation of food consumption and/or energy expenditure^12^. **l**-**Trp** and **l** **-Phe** ^9, 16^ activate GPR139, which has therefor been propsed to be a nutrient-sensing receptor^9, 16^. In support of this hypothesis, the closest homolog GPR142 is also activated by **l**-**Trp** and **l** **-Phe** ^17, 18^ and activation of this receptor has been shown to lower blood glucose levels and increase insulin secretion in mice^17, 19, 20^ making it a new putative target for treatment of diabetes. Since the GPR139 receptor shares the same ligands and is expressed in the hypothalamus it is possible that GPR139 is also involved in the pathophysiology of diabetes. Furthermore, we have very recently shown that the endogenous POMC derived peptides ATCH, α-MSH, and β-MSH, known to be involved in energy homeostasis, also activate GPR139 *in vitro* ^21^. Taken together GPR139 has been hypothesized as a potential target for the treatment of the metabolic syndrome e.g. diabetes and eating disorders.

**Figure 1:**
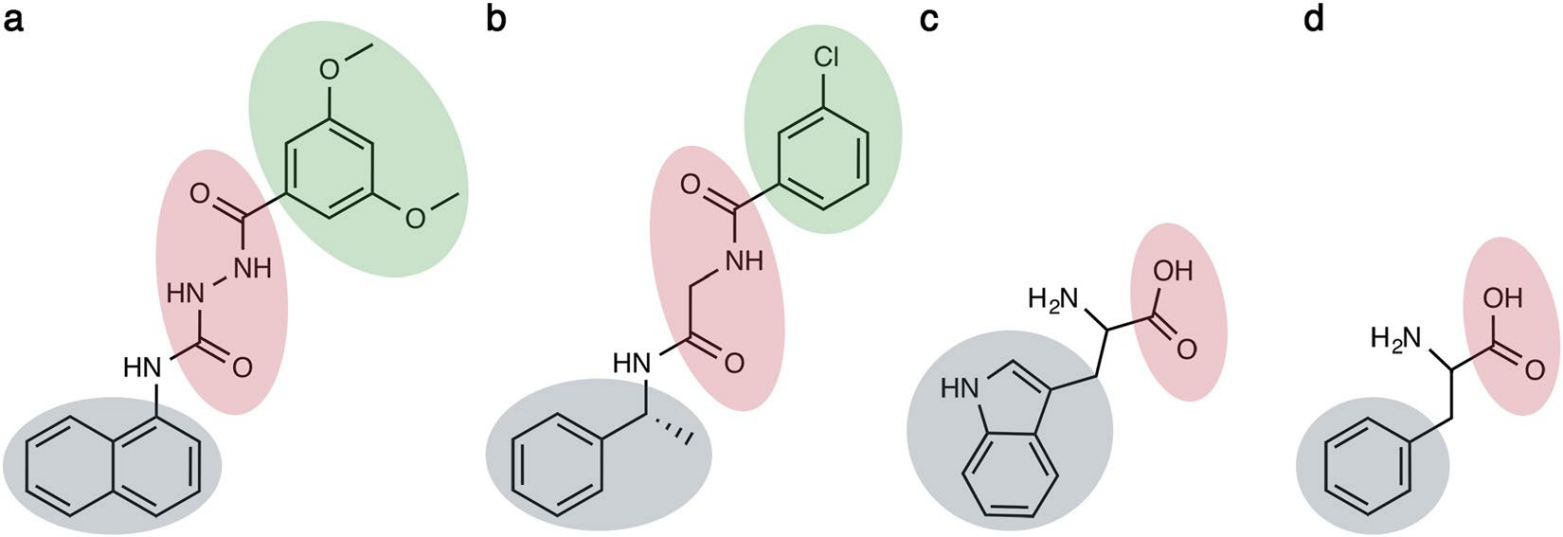
Structure of the GPR139 agonists studied herein. (**a**) Surrogate agonist **1a** from Shi *et al*.^23^. (**b**) Surrogate agonist **7c** from Dvorak *et al*.^14, 24^. (**c**,**d**) **Trp** and **Phe** from Isberg *et al*.^16^. Coloring denote chemical commonalities (supported by mutations herein); grey: major hydrophobic part, red: polar linkers (**1a** and **7c**) or carboxyls (**Phe** and **Trp**), green: hydrophobic element unique for the larger **1a** and **7c**.

Besides the natural aromatic amino acids **l**-**Trp** and **l** **-Phe** ^9, 16^ and the endogenous POMC derived peptides^21^, GPR139 has been reported to bind surrogate small molecules (e.g. **1a** and **7c**)^13, 22–26^. In the present study, an initial homology model of GPR139 in complex with **1a** was used to guide a site-directed mutagenesis (SDM) study of the GPR139 ligand binding site, which was performed on ligands **1a**, **7c**, **l**-**Trp** and **l** **-Phe**, bearing a common structure-activity relationship (SAR) profile as shown earlier^26^ and summarized in Fig. 1. The mutations and binding modes were further examined *in silico* using a ligand-steered homology modeling approach^27^, in this case based on cycles of molecular dynamics (MD) and free energy perturbation (FEP) on selected alanine mutations^28–30^ used as a scoring function. From these results, a common interaction profile for the selected GPR139 agonists was confirmed.

## Results

### Mutations selected from a preliminary 1a binding mode model

Docking of compound **1a** in our preliminary GPR139 structure model indicated four residues; E105^3×29^, E108^3×32^, N271^7×38^ and R244^6×51^ with putative hydrogen bonds to the ligand linker (Supplementary Fig. 1a). The **1a** naphthyl ring displayed a tight fit inside a deep hydrophobic pocket lined by F109^3×33^, H187^5×43^, and W241^6×48^. Binding poses with a flipped ligand orientations were also seen. However, the above-described pose got the highest score (−9.51 compared to −5.29) and is the only one that agrees with the mutation data. Therefore, we decided to only move forward with that and similar poses. Based on this binding pose, we selected 18 binding site mutations (of 12 residues) for pharmacological testing (Supplementary Fig. 1b). The mutations focused solely on residues in the transmembrane region, as none of the extracellular loops were in proximity of the ligand binding site.

### Surface expression of GPR139 mutants

**Figure 2:**
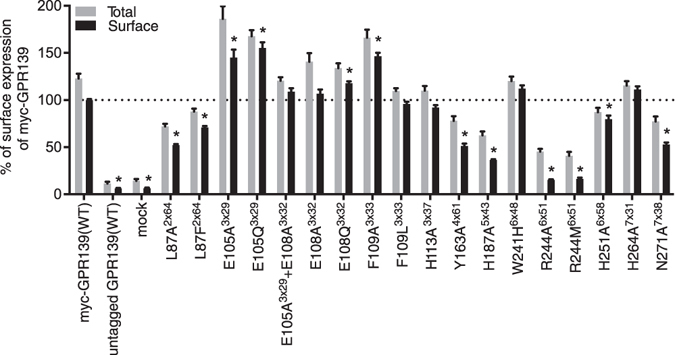
Total and surface expression of GPR139 mutants. Cell surface expression profiles of each of the human GPR139 mutants compared to myc-GPR139(WT). Grey bars = total expression (triton-X treated), black bars = surface expression. Data is mean ± S.E.M. of 4–8 independent experiments performed in triplicates. Statistical analysis was performed on the surface expression using one-way ANOVA followed by Dunnett’s post-hoc test in comparison with the surface expression of myc-GPR139(WT) (*P < 0.01).

Cell surface expression was measured with ELISA using a myc-tag positioned at the extracellular N-terminal of GPR139. The myc-tagged wild-type (WT) receptor displayed equivalent response to **1a** in the Fluo-4 Ca^2+^-assay (EC_50_ = 670 nM) as compared to the untagged WT receptor (EC_50_ = 772 nM) (Supplementary Fig. 2). Both R244^6×51^ mutants (alanine and methionine) lost surface expression, while the expression of H187A^5×43^ was significantly reduced (36% of WT, Fig. 2). Modestly reduced surface expression (>50%) was observed for the mutants L87A^2×64^, L87F^2×64^, Y163A^4×61^, and N271A^7×38^ whereas the remaining mutants showed WT-like or even increased surface expression compared to WT (Fig. 2).

### The 1a binding site

#### *In vitro* mutation effects on ligand potencies

**Figure 3:**
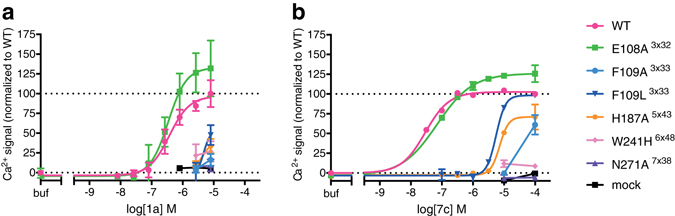
Effects of GPR139 mutations on pharmacological profiles of 1a and 7c. The data demonstrates that the residues F109^3×33^, H187^5×43^, W241^6×48^ and N271^7×38^ are important for GPR139 activation by **1a** and **7c**, whereas residue E108^3×32^ is not. Concentration-response curves of (**a**) **1a** and (**b**) **7c**, on the mutants with an effect (plus WT, mock and E108A^3×32^). The graphs are one representative (mean ± S.D.) out of three independent experiments performed in (**a**) triplicates and (**b**) duplicates. All responses are normalized to myc-GPR139(WT) (0% = buffer, 100% = 8 μM **1a** or 100 μM **7c**).

The ability of **1a** to activate the mutant receptors was measured in a Fluo-4 Ca^2+^-assay (Supplementary Fig. 3). Complete loss-of-function was observed for mutants F109A^3×33^, F109L^3×33^, and N271A^7×38^, while **1a** displayed markedly reduced potency at W241H^6×48^ and H187A^5×43^ (Fig. 3). Noteworthy, for the H187A^5×43^ and N271A^7×38^ mutants their lower expression levels (36% and 53%, respectively) could also have contributed to the reduction in potency. However, **1a** was equipotent on L87A^2×64^ indicating that mutants with reduced expression can sometimes still induce a normal functional response. All other mutations showed no significant (>10-fold) change in potency, indicating that these residues are not critical for **1a** activity (Table 1 and Supplementary Table 1).

**Table 1:** GPR139 mutant potencies for **1a**, **7c**, **l**-**Trp**, and **l** **-Phe**.

Mutant	% SE	1a		7c		l-Trp		l-Phe	
		pEC_50_ ± SEM	E_max_ ± SEM	pEC_50_ ± SEM	E_max_ ± SEM	pEC_50_ ± SEM	E_max_ ± SEM	pEC_50_ ± SEM	E_max_ ± SEM
WT	100	6.63 ± 0.08	100	7.32 ± 0.16	100	3.70 ± 0.14	100	3.43 ± 0.22	100
E108A^3×32^	109	6.68 ± 0.11	151 ± 9	6.82 ± 0.25	112 ± 10	3.78^§^	125	3.60^§^	127
F109A^3×33^	146	<5.1	NE*	<4.0	60 ± 3**	<2	60 ± 10^#^	<1.5	60 ± 6^##^
F109L^3×33^	96	<5.1	42 ± 16*	5.40 ± 0.07	102 ± 3	<2	60 ± 5^#^	<1.5	72 ± 9^##^
H187A^5×43^	36	<5.1	35 ± 5*	5.18 ± 0.06	60 ± 9	ND	ND	ND	ND
W241H^6×48^	112	<5.1	27 ± 10*	<4.0	12 ± 3**	<2	NE^#^	<1.5	NE^##^
N271A^7×38^	53	<5.1	NE*	<4.0	NE**	<2	31 ± 5^#^	<1.5	38 ± 9^##^

The table displays the mutants with effect on **1a** (and E108A^3×32^), and a percent surface expression (% SE, normalized to WT = 100%) over 35%. The potencies are presented as mean pEC_50_ ± SEM and mean E_max_ ± SEM. The potency of **1a** was calculated from three independent experiments conducted in triplicates, and normalized to buffer (0%) and 8 μM **1a** (100%). The potencies of **7c**, **Trp** and **Phe** are from three independent experiments conducted in duplicates, and normalized to buffer (0%) and 100 μM **7c** or 10 mM **Trp** or 30 mM **Phe** (100%), respectively. Remarks: *at 8 μM, **at 100 μM, ^#^at 10 mM, ^##^at 30 mM, ^§^only one experiment, ND: not determined (not measured) and NE: no effect (loss of activity).

#### In silico mutation effects on calculated binding affinities

The initial GPR139-**1a** structural model described above was optimized to take into account the mutagenesis data (see methods). This was done by means of iterative MD simulations, coupled to free energy perturbation (FEP) calculations as a scoring function (Supplementary Figs 4 and 5). The three mutations to alanine that showed a significant effect and were sufficiently expressed were chosen for scoring, i.e. residues F109A^3×33^, H187A^5×43^ and N271A^7×38^. Mutation W241H^6×48^ also showed drastic effects on ligand potency, but the dynamic role of this residue (activation switch in class A GPCRs^31^) precluded us from using the data for its mutation to histidine in our model optimization.

The calculated difference in binding free energies for the mutant receptors in the first and second iterations did not correlate well with the experimental mutagenesis data, and the analysis of the trajectories showed unstable protein (iteration 2) and ligand (iteration 1 and 2) conformations. The third MD/FEP iteration showed significant effects for mutants F109A^3×33^ and N271A^7×38^, while the H187A^5×43^ displayed a large standard error of the mean (s.e.m.) (Table 2). For this reason, we ran a final iteration consisting of re-docking and MD/FEP of **1a** in the conformation of GPR139 obtained after a similar iteration with ligand **7c** (see next section). The obtained final model reproduced the *in vitro* potency data of **1a** by showing positive contributions to the binding free energies (which corresponds to lower ligand affinity) and showed that the FEP scoring approach was able to distinguish between low (iteration 1) and high (iteration 4) quality models.

**Table 2:** GPR139 *in silico* mutant effects of **1a** and **7c** binding.

Mutation	Change of *in vitro* potency		*In silico* relative binding free energies (ΔΔG kcal/mol)				
	1a	7c	1a			1a	7c
			Iteration 1	Iteration 2	Iteration 3	Final model	Final model
F109A^3×33^	Loss of function	>2000 fold decreased	−3.14 ± 0.84	0.66 ± 2.02	**1.25 ± 1.03**	**6.52 ± 0.87**	**6.27 ± 0.66**
H187A^5×43^	>34 fold decreased	138 fold decreased	−3.03 ± 1.04	−1.26 ± 1.60	0.56 ± 1.11	**2.64 ± 0.93**	**1.72 ± 1.29**
N271A^7×38^	Loss of function	Loss of function	0.25 ± 0.47	**4.40 ± 0.54**	**3.73 ± 0.29**	**3.21 ± 0.45**	**2.71 ± 0.34**

Iterative procedure of model optimization using molecular dynamics and the relative binding free energies obtained by free energy perturbation (FEP) calculations in comparison with *in vitro* potency as a scoring function. The FEP relative binding free energies that are in agreement with *in vitro* data are shown in bold.

#### Binding mode in the receptor model

**Figure 4:**
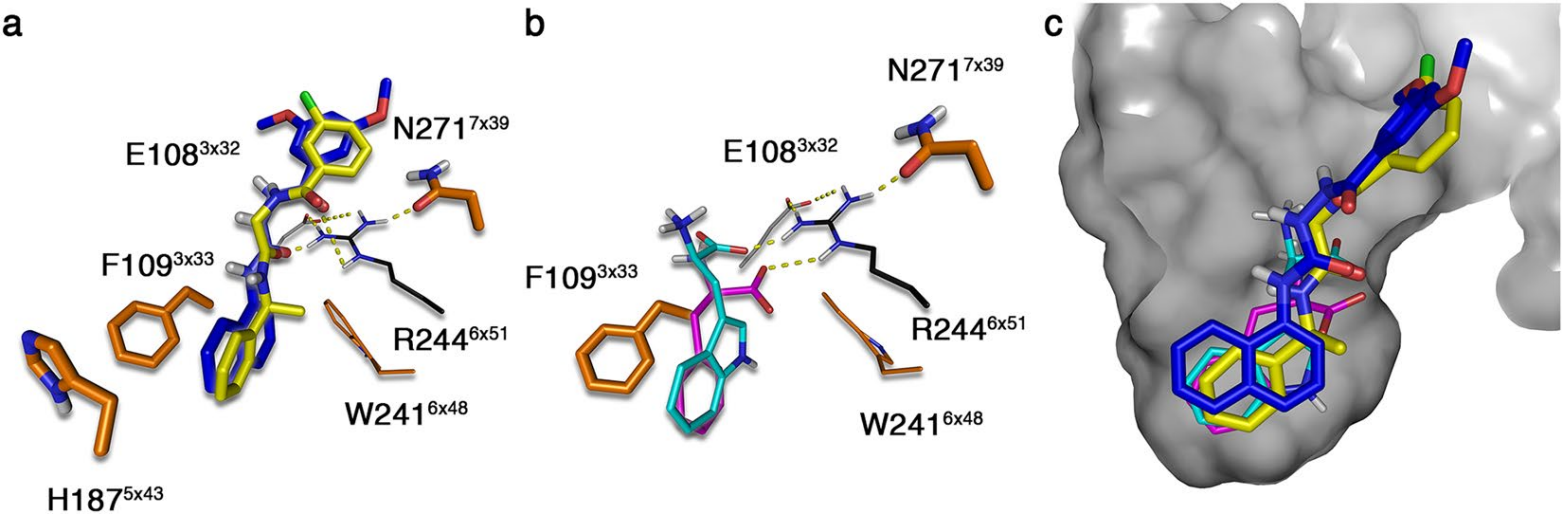
**1a**, **7c**, **l**-**Trp**, and **l** **-Phe** binding pose models. (**a**) Binding mode of **1a** (blue) and **7c** (yellow) and (**b**) endogenous amino acids **l**-**Trp** (cyan) and **l** **-Phe** (magenta). Mutations that showed a significant effect when mutated are colored orange. Residues with thick sticks have been mutated *in silico* and *in vitro* (F109^3×33^, H187^5×43^ and N271^7×39^) and those with thin sticks *in vitro* only (W241^6×48^). The latter was excluded due to the dynamic role of this residue as an activation switch in class A GPCRs^25^. Residues colored in grey showed no significant changes in potency (E108^3×32^) and those in black were not expressed respectively (R244^6×51^). (**c**) Overlay of all four studies ligands within the GPR139 binding pocket shown as a surface. All tested agonists bind a deep hydrophobic pocket and are shown to undergo hydrogen bonding with R244^6×51^.

The **1a** naphthyl ring was positioned in a deep hydrophobic pocket lined by F109A^3×33^, H187A^5×43^, and W241H^6×48^ (Fig. 4a); all of which displayed significant effects upon mutation. The available SAR for **1a** confirms tight binding of the naphthyl ring, as substitution in the 4, 5 or 7 positions abolishes ligand binding affinities^23^. The linker in **1a** displayed hydrogen bonds to N271^7×38^ and R244^6×51^. Notably, the model did not show a hydrogen bond to E108^3×32^, but instead an indirect interaction via R244^6×51^. This is in agreement with the mutation data that showed no effect for E108A^3×32^ and a modest 6-fold potency reduction for E108Q^3×32^, in which the carboxamide nitrogen may have unfavorable contact with R244^6×51^.

### The 7c binding site

#### *In vitro* mutation effects on ligand potencies

All mutants that had an effect on **1a** also affected **7c** potency, although F109L^3×33^ and H187A^5×43^ displayed a milder (yet ~100-fold) effect (Table 1).

#### In silico mutation effects on calculated binding affinities

Compound **7c** was docked in the optimized structure of GPR139 obtained from iteration 3 of the GPR139-**1a** complex, resulting in similar poses for the two ligands. However, during the equilibration phase of the subsequent run of MD/FEP the ligand **7c** readjusted its initial pose to bind deeper in the binding pocket. This resulted in a stable conformation that gives calculated energies in excellent agreement with the *in vitro* results for this ligand (Table 2). Thus, we re-docked **1a** in the resulting structure of GPR139 and ran a final (fourth, see above) MD/FEP iteration for this ligand, obtaining excellent qualitative agreement with the experimental data as outlined above (Table 2). The two ligands thus show very similar effects for each of the three mutations considered both *in vitro* and *in silico*. Notably, the experimental data on ligand potency for **7c** shows a somewhat milder effect for H187A^5×43^ than F109A^3×33^ and N271A^7×38^, which is in line with the lower calculated effect of the H187A^5×43^ mutation.

#### Binding mode in receptor model

The final poses for **1a** and **7c** overlap nearly perfectly (common scaffold RMSD 0.529 Å, Fig. 4a) and they display nearly identical receptor interactions. This reflects their high similarity in both the *in vitro* and *in silico* mutants. This confirms a common binding mode for these two ligands.

### The l-Trp and l-Phe binding site

#### *In vitro* mutation effects on ligand potencies

The mutations displayed very similar effects on the amino acids **l**-**Trp** and **l** **-Phe** as for **1a** and **7c** (Table 1 and Supplementary Fig. 6).

#### Binding mode in receptor model

Both amino acids showed a similar binding mode (Fig. 4b), placing the hydrophobic functional groups of the amino acids deeply in the binding pocket in overlay with hydrophobic moieties of **1a** and **7c** (Fig. 4c). Furthermore, the charged backbone functionalities overlap with the polar linkers of **1a** and **7c**.

## Discussion

We identified a common binding site for the GPR139 surrogate agonists; **1a**, **7c** and the endogenous amino acids **l**-**Trp** and **l** **-Phe** (Fig. 4). The proposed binding pocket consists of a deeply buried hydrophobic region between F109^3×33^ H187^5×43^ and W241^6×48^, and a polar region defined by N271^7×38^, R244^6×51^ and E108^3×32^. The four ligands position themselves in a way that all have an aromatic moiety in the deep hydrophobic region, while their polar (**1a** and **7c**) or charged (amino acids) regions bind to N271^7×38^ and R244^6×51^. This is supported by the loss of ligand activity in analogs lacking one of these carbonyls^23, 24^. E108^3×32^ is indicated to have only indirect interaction via R244^6×51^.

The binding mode matches our recent 3D pharmacophore of different series of GPR139 agonists^16, 26^. One of the two hydrophobic pharmacophore elements harbors the same ligand moieties (**1a** naphthyl, **7c** phenyl and **l** **-Phe**/**l**-**Trp** sidechains) deep in the hydrophobic part of the binding site. R244^6×51^ and pharmacophore hydrogen bond acceptor elements both match the polar linkers (**1a** and **7c**) or charged amino acids backbones. The second terminal phenyl in **1a** and **7c** points to the top of TM2, in contact with Phe83^2×60^ and Leu86^2×63^. A hydrogen bond acceptor functionality in this ring assigned by the pharmacophore model could correspond with the interaction of the methoxy in **1a** or the chloride atom in **7c** with N271^7×38^.

FEP calculation dependence of the use of starting structure was traditionally considered a limiting factor for the widespread application of this technique. However, current computational power and the increased robustness of the protocols have made it possible to use the FEP results as a filter to discriminate the most reliable binding mode from a pool of solutions^28^. In this work, we applied FEP to refine an initial binding mode in an iterative fashion. This iterative protocol allowed for the identification of a binding mode that correlates with and provides satisfactory explanation to the experimental data. As shown in iteration 1, this effect could not be captured with a more simple and intuitive use of docking and modeling.

## Conclusions

Our combined *in vitro* and *in silico* mutagenesis demonstrates that residues F109^3×33^, H187A^5×43^, and N271^7×38^ are highly important for all studied ligands. We provide (Supplementary Data) highly refined GPR139-ligand complex structure models that fully agree with these data and may serve to guide future mutagenesis or ligand design, including identification of much needed antagonists with increased potency and selectivity. Hence, this first report of the GPR139 binding site paves the way for future studies towards the characterization of GPR139 pharmacology and function.

## Methods

### Receptor mutagenesis

To study the influence of specific amino acids in the predicted binding pocket on receptor function, the desired mutations were introduced into an N-terminally c-myc-tagged wild type human GPR139 (NM_001002911.3) in the pEGFP-N1 vector (BD Biosciences)^32, 33^. As previously described^33^, these plasmids carry the signal peptide of mGluR5 to promote cell surface expression followed by a c-myc epitope to enable detection of total and surface expression by ELISA, followed by an engineered MluI and NotI site for easy insertion of the receptor of interest. Furthermore, the start-codon in the GPR139 sequence was deleted to ensure c-myc-tagging. Mutagenesis was designed in Serial Cloner 2.6 (© Franck Perez [SerialBasics]) and carried out by GenScript (USA). All mutated residues are illustrated in Supplementary Fig. 1b. An untagged GPR139(WT) in pEGFP-N1 was also generated to ensure that the c-myc-tag had no influence on GPR139(WT) function.

### Transfection and cell culture

All GPR139 mutants were transfected into HEK293 cells. The HEK293 cells were grown in Dulbecco’s modified Eagle’s medium (DMEM) (Gibco, 41965) supplemented with 10% dialyzed fetal bovine serum (Gibco, 26400, United States origin), 100 units/mL penicillin and 100 μg/mL streptomycin (Gibco, 15140). HEK293 cells were reverse transfected using Lipofectamine2000 (Invitrogen). More specifically two mixtures were made: 1) 10 ng DNA/well + 25 μL Opti-MEM/well and 2) 250 μg Lipofectamine2000/well + 25 μL Opti-MEM/well. The two mixtures were mixed and 50 μL/well was added directly in black 96-well plates with flat clear bottoms for the Fluo-4 Ca^2+^-assay (Corning, Falcon, 353219) or in white Opaque 96-well Microplate (PerkinElmer Life, 6005680) for ELISA. 100 μL containing 20,000 cells were added to each well and incubated 48 hours before assays. All plates were coated with poly-d-lysine.

### ELISA

Cells were fixed using 50 μL/well 4% paraformaldehyde for 5 minutes (min). The wells were washed twice with DPBS (Gibco, 14190) containing 1 mM Ca^2+^ (DPBS-Ca), followed by addition of either 50 μL/well 0.1% triton-X (for detection of total expression) or 50 μL/well DPBS-Ca (for detection of surface expression) for 5 min. The wells were again washed twice with DPBS-Ca, followed by the addition of 100 μL/well blocking solution (ddH_2_O with 3% skim milk, 1 mM Ca^2+^, 50 mM Trizma hydrochloride solution pH 7.4) and incubated at room temperature (RT) for 30 minutes. After blocking, 75 μL/well 1:1000 c-myc mouse monoclonal antibody (Invitrogen, R950-25) diluted in blocking solution was added to each well and incubated at RT for 45 minutes. Subsequently, each well was washed with 100 μL/well blocking solution and twice with 100 μl/well DPBS-Ca. Then 75 μL/well 1:1500 sheep anti-mouse IgG HRP conjugate (VWR, NA931-100UL) diluted in blocking solution was added, and incubated at RT for 45 minutes. The wells were then washed four times with 100 μL/well blocking solution and four times with 100 μL/well DPBS-Ca. At the end 65 μL/well DPBS-Ca was added, and the detection solution was prepared (SuperSignal ELISA Femto stable peroxidase solution and SuperSignal ELISA Femto luminol enhancer solution (Thermo Fisher Scientific, Waltham, MA), 1:1); 15 μL/well detection solution was added to the plate, and chemiluminescence was measured immediately on an EnSpire reader (PerkinElmer Life and Analytical Sciences).

### Ligands

Compound 1a (**1a**) was kindly provided by H. Lundbeck A/S, Denmark. Compound **7c** (Enamine, Z31449867) was tested as a racemate, as the (S)-form described by Janssen *et al*. was not commercially available. Both compounds were dissolved to 20 mM in DMSO (Sigma, D2650) and subsequently diluted in a HEPES buffer (HBSS (Gibco, 14025) supplemented with 20 mM HEPES + 1 mM MgCl_2_ + 1 mM CaCl_2_, pH = 7.4) to a final concentration of 0.5% DMSO in the Fluo-4 Ca^2+^-assay. The DMSO level was kept constant for all concentrations of both compounds. DMSO was confirmed not to have any activity by itself at this concentration^16^. **l**-**Trp** (T0254) and **l** **-Phe** (P2126) were obtained from Sigma-Aldrich and dissolved in buffer.

### Fluo-4 Ca^2+^-assay

The Ca^2+^ measurements were preformed using the Fluo-4 NW Calcium Assay Kit (Invitrogen, Molecular Probes, F36206) as previously described^26^. Briefly, the Fluo-4 dye loading solution was prepared according to the manufacturer’s instructions by dissolving it in HEPES-buffer supplemented with 2.5 mM probenecid. 50 μL dye loading solution was added to each well. Cells were incubated with the Fluo-4 dye for 60 min at 37 °C, then washed with 100 μL HEPES-buffer. 100 μL HEPES-buffer supplemented with 2.5 mM probenecid was then added to each well and incubated in 10 minutes at 37 °C before measurement. 33 μL of **1a**, **7c**, **l**-**Trp** or **l** **-Phe** (4x concentrated) were added automatically after baseline measurements. Intracellular calcium changes were recorded as indicated on either a NOVOstar (BMG Labtech) at 37 °C with an excitation filter of 485 nm and an emission filters 520 nm or a FlexStation 3 Benchtop Multi-Mode Microplate Reader (Molecular Devices) at 37 °C with an excitation wavelength of 485 nm and emission of 525 nm.

### Data analysis and statistics

All pharmacological data have been analyzed by using Prism 6.0 (GraphPad Software Inc., San Diego). Fitting of concentration response curves was performed by non-linear regression log(agonist) vs. response (four parameters). Pooled data are shown with standard error of the mean (S.E.M.) and data showing one representative are shown with standard deviation (S.D.). Changes in potency (pEC_50_) and span of myc-GPR139(WT) in comparison to untagged GPR139(WT) was statistically analyzed with a paired t-test and significance was accepted at p < 0.05. The ELISA data were normalized to surface expression of myc-GPR139(WT) (100%). The changes in cell surface expression of GPR139 mutants in comparison with WT control and the changes in potency of GPR139 mutants in comparison with WT control were statistically analyzed with one-way analysis of variance (ANOVA) and Dunnett’s post-hoc test.

### Homology modeling

The crystal structure of the active human serotonin 2B receptor (5HT_2B_ - PDB: 4IB4)^34^ was selected as an initial template using the online GPCRdb template selection tool^35, 36^. The structure has a resolution of 2.7 Å, and the protein sequence similarity between GPR139 and the template structure is 42% in the seven trans-membrane helical (7TM) region. The protein sequences of the template and target were aligned with MEGA6^37^, while utilizing GPCRdb sequence alignment and the main template as a reference for assigning the seven helical tips. The overall receptor structure was built with Modeller9 v.13^38^, and loops further refined with the LoopModel routine therein implemented. In general, a disulphide bridge between ECL2 Cys^45×50^ and Cys^3×25^ is a conserved feature among most Class A GPCRs. However, this feature was not incorporated in our GPR139 model, as both ECL2 and TM3 lack a Cys residue in those positions. To enhance the GPR139 model for subsequent ligand docking, the rotamers of residues that are not conserved between the target and template were manually defined based on the most homologous template from an in-house GPCR position-specific rotamer library that contains rotamers extracted from all published GPCR crystal structures^39^. Therefore the rotamers of the following residues were refined: Y33^1×39^, L36^1×42^, L86^2×63^, E105^3×29^, F109^3×33^, I184^5×40^, W241^6×48^, I248^6×55^, H251^6×58^, H264^7×31^, D268^7×35^, N271^7×38^, and L275^7×42^. All rotamers were collected from our in-house GPCR position-specific rotamer library^39^. On the other hand, the rotamer of R244^6×51^ – a critical amino acid in the binding site with no available rotamers in our library – was refined based on the rotamer library available in Maestro (Schrödinger Release 2015-3)^40^. In total, 12 of 18 residues (~67%) were refined in accordance with a similar crystal structure template. The quality of the model was assessed by Ramachandran plots within the PROCHECK webserver^41^.

Another homology model based on the inactive human opioid kappa receptor (OPRK - PDB: 4DJH)^42^ was also built. The template structure has a resolution of 2.9 Å, and an overall 7TM similarity of 42% to GPR139. The model was built and its quality assessed using the same software and routines as described above.

### Ligand Docking and Molecular Visualization

The two homology models were prepared for docking studies with the Schrödinger Protein Preparation Wizard, including a hydrogen optimization at pH = 7 of the ionisable polar groups using Maestro PROPKA^43^.

Docking was done with Glide^44–46^ with default settings. The partial charges of the ligand were assigned by Epik^47, 48^ using the OPLS_3 force field. Further options were set to allow the rotation of hydroxyl hydrogen atoms in the binding site, enhance the planarity of conjugated π-systems, and to include the Epik state penalties to the scoring calculations. Flexible ligand sampling was applied combined with biased sampling of amide groups (penalization of nonplanar conformations). The docking grid centroid was placed around binding pocket residues E105^3×29^, F109^3×33^, W241^6×48^, and R244^6×51^, and the cubic grid box sides were set at 10 Å. Subsequently, the resulting receptor–ligand complexes were minimized using the energy minimization tool in MacroModel Schrödinger^49^. The TNCG (Truncated Newton Conjugate Gradient) minimization method was used with maximum iteration steps set to 5000, and with a convergence gradient of 0.001. Heavy atoms were strained at a ±0.3 Å radius while applying a force constant of 120 kcal/mol/Å^2^. Poses correlating with the mutation data were used for further MD analysis. All 3D images were produced in PyMOL^50^.

### Membrane insertion and equilibration

Ligand receptor complexes obtained in the previous stage were inserted in the membrane and equilibrated under periodic boundary conditions (PBC) using the PyMemDyn protocol described elsewhere^51^. Shortly, the starting structure is automatically embedded in a pre-equilibrated membrane consisting of POPC (1-palmitoyl-2-oleoyl phosphatidylcholine) lipids, with the TM bundle aligned to its vertical axis. This hexagonal-prism shaped box is then soaked with bulk water and energy minimized with GROMACS 4.6^51, 52^, using the OPLS-AA force field^53^ for protein and ligands, combined with the Berger parameters for the lipids^54^. The same setup is used for a 2.5 ns MD equilibration, where initial restraints on protein and ligand atoms are gradually released as described in detail in ref. 51.

### MD and FEP calculations

The MD software Q^55^ was used for free energy perturbation (FEP) calculations under spherical boundary conditions, using a 25 Å sphere centered on the center of geometry of the ligand. Protein atoms in the boundary of the sphere 22–25 Å outer shell) had a positional restraint of 20 kcal/mol/Å^2^, while solvent atoms were subject to polarization and radial restrains using the surface constrained all-atom solvent (SCAAS)^56, 57^ model to mimic the properties of bulk water at the sphere surface. Atoms lying outside the simulation sphere are tightly constrained (200 kcal/mol/Å^2^ force constant) and excluded from the calculation of non-bonded interactions. Long range electrostatics interactions beyond a 10 Å cut off were treated with the local reaction field method^58^, except for the atoms undergoing the FEP transformation where no cutoff was applied. Solvent bond and angles were constrained using the SHAKE algorithm^59^. The consideration of the binding site region under spherical boundary conditions offers several advantages over the alternate periodic boundary conditions for free energy calculations. Besides the obvious reduction in system size, it avoids possible larger scale conformational fluctuations distal to the binding site. Such fluctuations commonly introduce noise and decrease convergence for the free energy calculations, while a spherical system still considers the relevant fluctuations in the binding site, as previously demonstrated^60^. In the particular case of membrane proteins, we have recently reported a similar effect when increasing the sphere size to consider distal parts of the receptor and membrane environment^26^.

All titratable residues outside the sphere were neutralized and protonation states of the histidines were manually assigned. Histidine residues H113^3×37^, H187^5×43^, H251^6×58^, and H264^7×31^ were assigned a hydrogen atom on the δ nitrogen and residues H132^3×56^, H137^34×54^, H180^3×36^, and H181^3×37^ on the ε nitrogen. Ligand and lipid parameters were obtained from the previous MD stage, whereas residue parameters were translated from the latest version of the OPLSAA force field^61^.

The sphere was equilibrated for 0.61 nanoseconds, where temperature was increased from 0.1 to 298 K whilst slowly removing a 25 kcal/mol/Å^2^ restraint on all atoms, and the protein-ligand interactions maintained through a distance restraint (15.0 kcal/mol/Å^2^ force constant for distance between 1.8 and 2.2 Å) for hydrogen bonds between carbonyl atoms in the ligand and hydrogen atoms in R244^6×51^ and N271^7×38^. These restraints were gradually removed during an additional 2 nanosecond equilibration MD, before the data collection period. This phase consisted of 14 replicates with different initial velocities and a 0.25 nanosecond unbiased equilibration period for each amino acid mutation, before applying the FEP protocol for amino acid mutations as previously published^28–30^. Briefly, a given mutation of any residue to alanine is divided in several smaller subperturbations to allow for a smoother transition between the end-states. Three steps are introduced for groups of atom (charge groups) starting with the group with the highest topological distance (number of atoms) from the protein backbone: (i) removal of partial charges per charge group, (ii) the introduction of a soft core van der Waals potential and (iii) the full annihilation of the atom(s). The last step includes the introduction of the Cβ hydrogen atom of the alanine residue. A mutation consists of eight, seven and five subperturbations of 51 λ windows of 10 ps each for phenylalanine, histidine and asparagine mutations, respectively. In the final models (iterations three and four) we tripled the simulation time to increase the convergence and hysteresis.

## Electronic supplementary material

Supplementary Information Supplementary Information
