# Supplementary material for: The GPR139 reference agonists 1a and 7c, and tryptophan and phenylalanine share a common binding site
Source: Sci Rep. 2017 Apr 25;7:1128. doi: 10.1038/s41598-017-01049-z (PMC5430874; doi:10.1038/s41598-017-01049-z)
Supplement: Supplementary file 1 — Supplementary Information [file 41598_2017_1049_MOESM1_ESM.pdf]

Supplementary Information

The GPR139 reference agonists 1a and 7c, and tryptophan and phenylalanine share a common binding site

Anne Cathrine Nøhr[1†], Willem Jespers[2†], Mohamed A. Shehata[1†], Leonard Floryan[3], Vignir Isberg[1], Kirsten Bayer Andersen[1], Johan Åqvist[2], Hugo Gutiérrez-de-Terán[2], Hans Bräuner-Osborne[1§]* and David E. Gloriam[1§]*

[1] Department of Drug Design and Pharmacology, University of Copenhagen, Denmark

[2] Department of Cell and Molecular Biology, Uppsala University, Biomedical Center, Box 596, SE-751 24, Uppsala, Sweden

[3] Department of Chemistry and Applied Biosciences, ETH Zürich, Switzerland

[†] Author's contributed equally, [§] Author's contributed equally

*Corresponding authors. E-mail address: david.goriam@sund.ku.dk (D.E.G. computational chemistry), hbo@sund.ku.dk (H.B.-O. pharmacology)

Contents

Supplementary Figure 1. Docking of 1a into the initial GPR139 model to suggest binding site residues.

Supplementary Figure 2. Myc-tagged GPR139(WT) and untagged GPR139(WT) have similar pharmacology.

Supplementary Figure 3. 1a on GPR139 mutants.

Supplementary Table 1. 1a activity on mutants that showed no significant change in potency that was 10-fold or more decreased.

Supplementary Figure 4. Flowchart of the binding site characterisation

Supplementary Figure 5. Investigated binding poses and correlating energies for 1a.

Supplementary Figure 6. Concentration-response curves of a) L-Trp and b) L-Phe on GPR139 mutants.

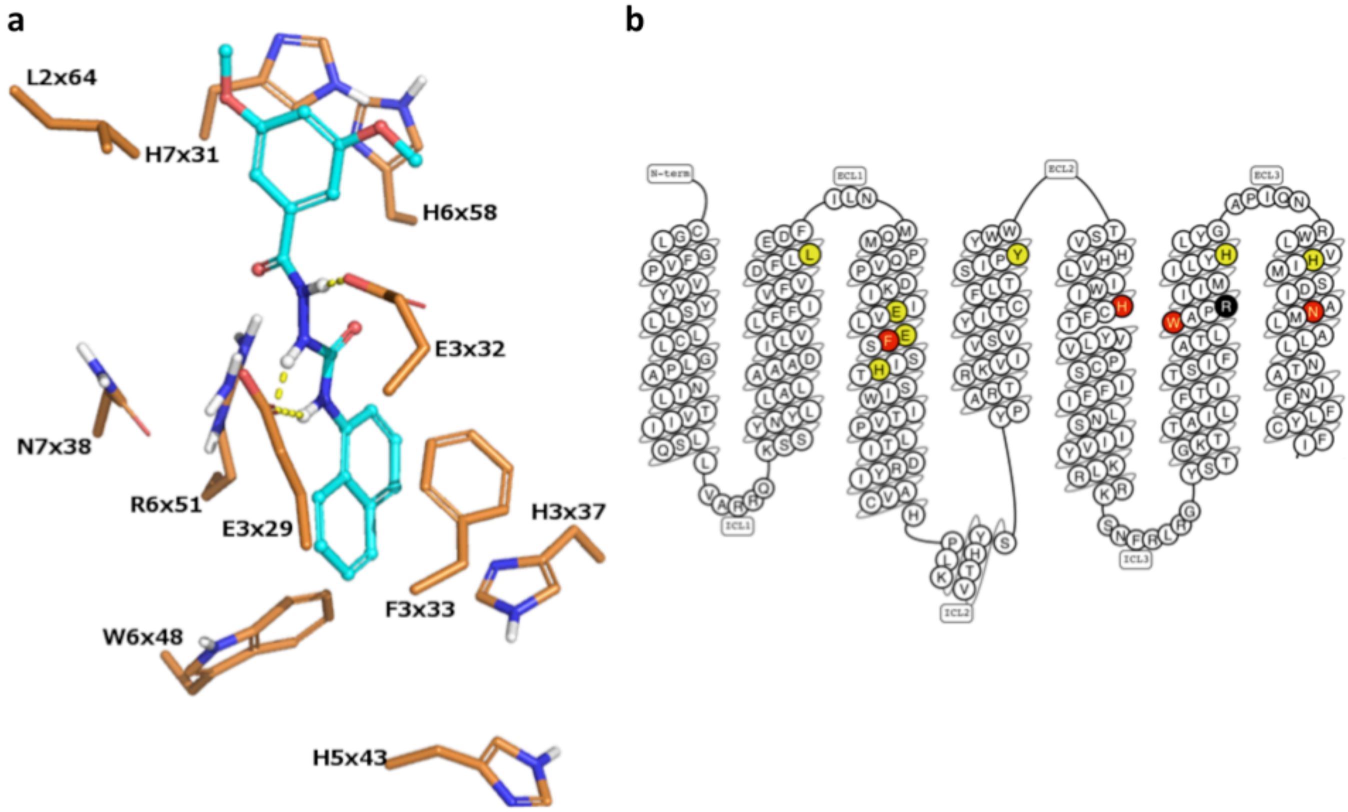

Supplementary Figure 1. Docking of 1a into the initial GPR139 model to suggest binding site residues. Twelve residues (18 mutations) in the predicted orthosteric binding site of GPR139 were chosen for mutagenesis studies. Top: **a**) **1a** (cyan) docked and potential interacting residues are highlighted (orange) **b**): Snake plot diagram[4,5] of GPR139 showing the twelve mutated residues in red (hot spot), yellow (no effect), and black (very low expression).

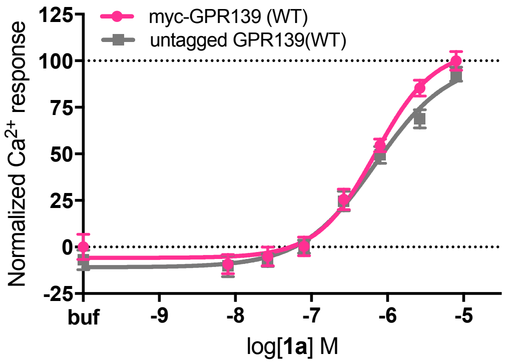

Supplementary Figure 2. Myc-tagged GPR139(WT) and untagged GPR139(WT) have similar pharmacology. The pharmacology of myc-GPR139(WT) and untagged GPR139(WT) was accessed measuring the Ca^{2+} response from the receptors when stimulating with **1a**. The Ca^{2+} response was normalized to the response from buffer (0%) and 8 μM **1a** (100%) on the myc-GPR139 (WT). All data are means ± SEM of three independent experiments conducted in triplicates on the NovoStar.

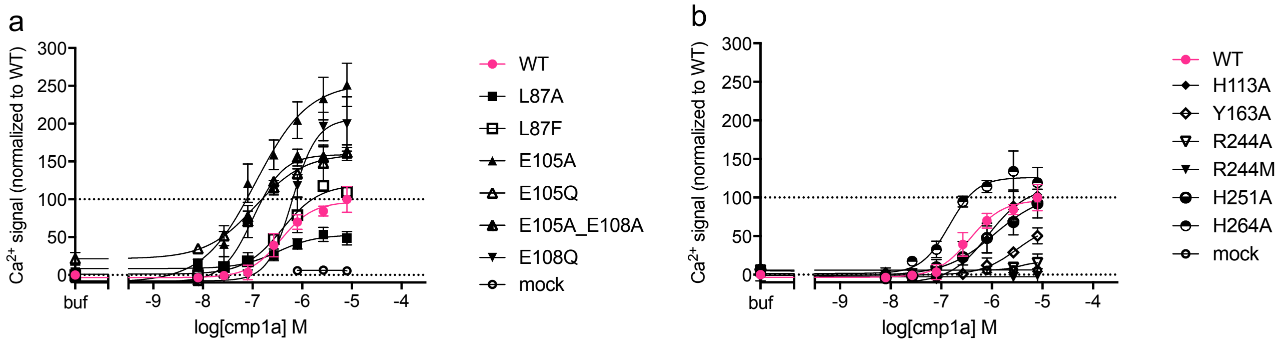

Supplementary Figure 3. 1a on GPR139 mutants. Concentration-response curves of **1a** on all GPR139 mutants. The graphs are one representative out of three independent experiments and are normalized to the response from buffer (0%) and 8 µM **1a** (100%). The data points are mean ± S.D. The results are summarized in table_invitro_all and Supplementary_Table_1a

**Mutant**	**% SE**	**1a**	
		**pEC_{50} ± SEM**	**E_{max} ± SEM**
**WT**	100	6.63 ± 0.08	100
**L87A^{2x64}**	52	6.32 ± 0.09	75 ± 17
**L87F^{2x64}**	71	6.37 ± 0.24	97 ±14
**E105A^{3x29}**	145	6.89 ± 0.15	288 ±27
**E105Q^{3x29}**	155	6.84 ± 0.13	177 ± 36
**E108Q^{3x32}**	107	5.82 ± 0.34	217 ± 21
**E108A^{3x29} +E105A^{3x32}**	118	6.70 ± 0.15	196 ±25
**H113A^{3x37}**	92	6.07 ± 0.03	97 ± 9
**Y163A^{4x61}**	51	<5.1	60 ± 10 *
**R244A^{6x51}**	15	not expressed	not expressed
**R244M^{6x51}**	16	not expressed	not expressed
**H251A^{6x58}**	80	6.09 ± 0.34	84 ± 11
**H264A^{7x31}**	111	6.60 ± 0.21	148 ± 15

Supplementary Table 1. 1a activity on mutants that showed no significant change in potency that was 10-fold or more decreased. SE = surface expression relative to WT (100%). The potencies are presented as mean pEC_{50} ± SEM and mean E_{max} ± SEM. The potency of **1a** are three independent experiments conducted in triplicates normalized to the response from buffer (0%) and 8 µM **1a** (100%). (*) at 8 µM, (NA) not applicable, since there was no response.

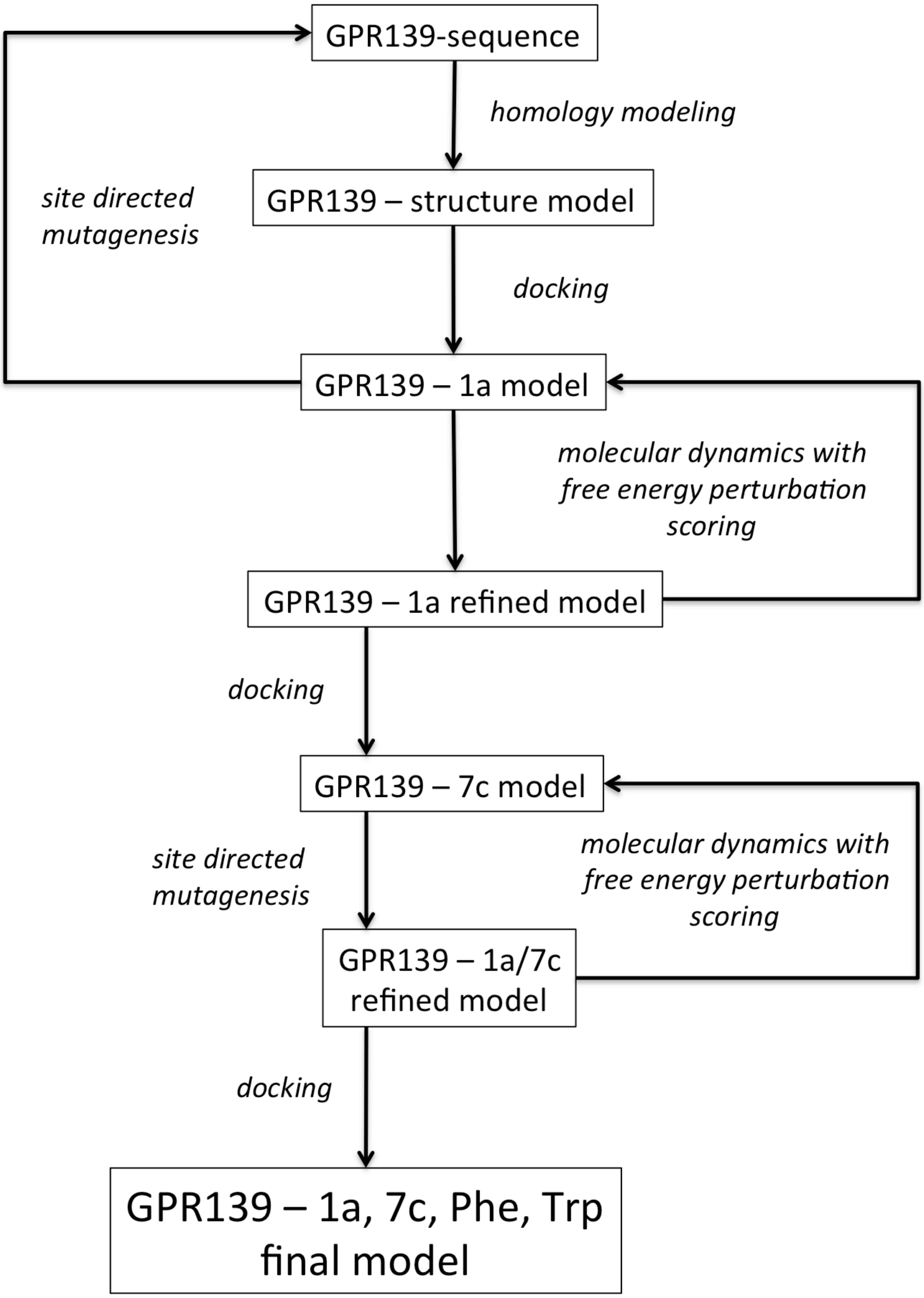

Supplementary Figure 4. Flowchart of the binding site characterisation. An initial homology model of GPR139 was created based on a 5-HT_{2B} template (PDB: 4IB4) and **1a** was docked into this model. Site directed mutagenesis (SDM) was done based on this model. A new GPR139 receptor model was built based on the obtained SDM data and a new more homologous template, the kappa-opoid receptor (PDB: 4DJH). An iterative approach of ligand-steered model optimisation was used with correlation of free energy perturbation shifts in binding affinity and SDM potency data as a scoring function for **1a**. The residues shown to be most important for **1a** binding were also tested on **7c** by experimental and computational mutagenesis. This final optimized model was validated using **1a, Phe** and **Trp**.

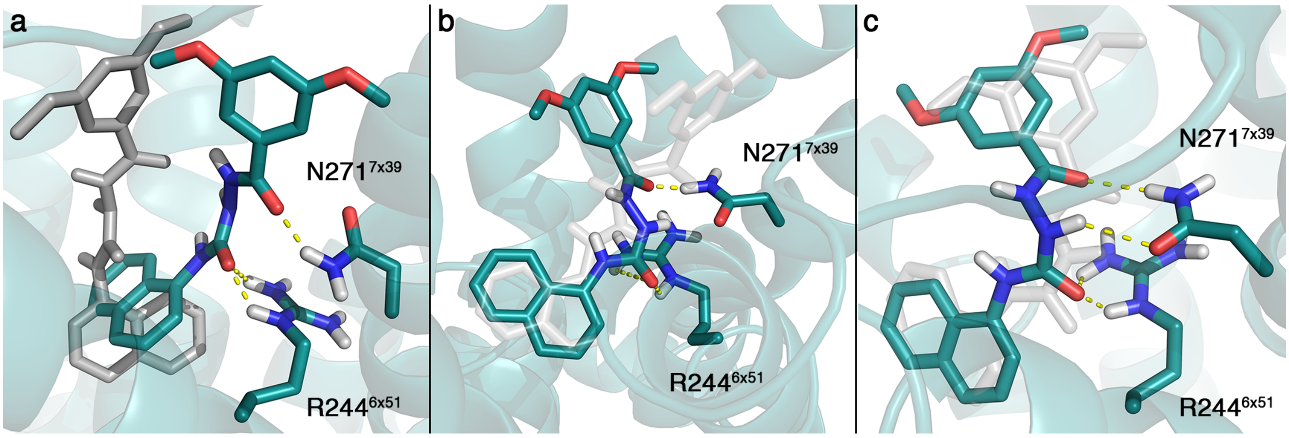

Supplementary Figure 5. Investigated binding poses and correlating energies for 1a. a) Initial docking based binding mode of **1a** (iteration 1 Table_XX_FEP). During equilibration **1a** moves away from R244^{6x51} and N271^{7x38} as a consequence of the downward movement of the naphthyl ring (grey). **b)** Redocked pose of **1a** (iteration 2 Table_XX_FEP) in grey the original docking pose. **c)** Ligand **1a** MD optimized pose after an extended equilibration (iteration 3 Table_XX_FEP). The ligand conformation in the binding pocket remained similar, indicating that the system was converged. Differences in binding affinity as compared to iteration 2 were caused by subtle changes in side chain conformation, solvation of the pocket and increased sampling.

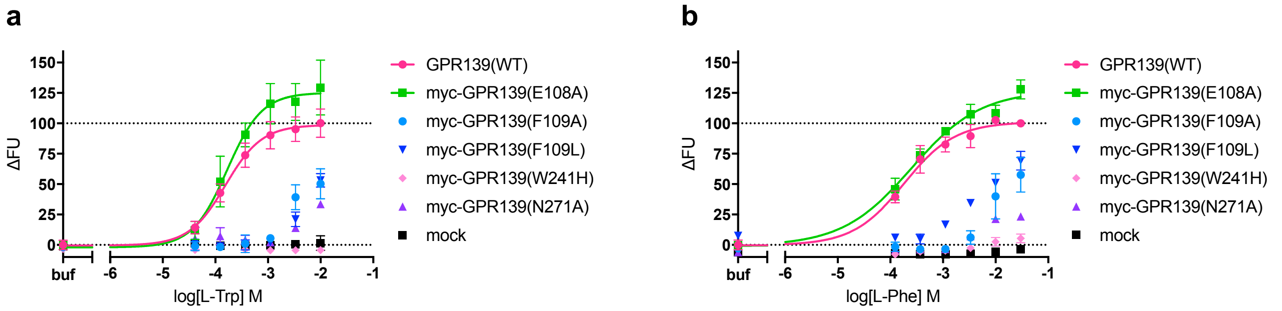

Supplementary Figure 6. Concentration-response curves of a) L-Trp and b) L-Phe on GPR139 mutants. The graph is one representative out of four independent experiments (except E108A n=1) performed in duplicates. All responses are normalized to the Ca^{2+} response of buffer (0%) or 10 mM L-Trp or 30 mM L-Phe (100%), respectively on myc-GPR139(WT).
